# Evaluation of Ocular Surface Health in Patients with Obstructive Sleep Apnea Syndrome

**DOI:** 10.4274/tjo.57778

**Published:** 2016-06-06

**Authors:** Emine Esra Karaca, Hanife Tuba Akçam, Feyzahan Uzun, Şengül Özdek, Tansu Ulukavak Çiftçi

**Affiliations:** 1 Sorgun State Hospital, Ophthalmology Clinic, Yozgat, Turkey; 2 Çankırı State Hospital, Ophthalmology Clinic, Çankırı, Turkey; 3 Recep Tayyip Erdoğan University Faculty of Medicine, Department of Ophthalmology, Rize, Turkey; 4 Gazi University Faculty of Medicine, Department of Ophthalmology, Ankara, Turkey; 5 Gazi University Faculty of Medicine, Department of Pulmonary Diseases, Ankara, Turkey

**Keywords:** Dry eye, obstructive sleep apnea syndrome, ocular surface health

## Abstract

**Objectives:**

To evaluate ocular surface health in obstructive sleep apnea syndrome (OSAS) and to investigate the tendency of these patients toward dry eyes.

**Materials and Methods:**

Fifty patients who underwent polysomnography and were diagnosed with OSAS and 50 normal control subjects were compared with respect to ocular surface disease index (OSDI), Schirmer I test and tear film break-up time (TBUT) values.

**Results:**

Patients were grouped as mild (n=15, 30%), moderate (n=15, 30%) and severe (n=20, 40%) according to apnea-hypopnea index values. The right eyes of patients were included in both groups. OSDI values were as follows: control group, 18.7±8.5; mild OSAS group, 40.2±2.8; moderate OSAS group, 48.5±2.2 and severe OSAS group, 62.7±2.3 (p<0.001). TBUT values were as follows: control group, 12.3±4.9; mild OSAS group, 8.2±4.7; moderate OSAS group, 5.8±2.1 and severe OSAS group, 4.2±3.7 (p<0.001). Schirmer values were as follows: control group, 18±6.1 mm; mild OSAS group, 12.9±6.7 mm; moderate OSAS group, 8.5±5.2 mm and severe OSAS group, 7.9±4.7 mm (p<0.001).

**Conclusion:**

Patients with OSAS seem to have a tendency toward dry eyes. Clinicians should be aware of dry eye development in these patients.

## INTRODUCTION

Obstructive sleep apnea syndrome (OSAS) is characterized by repeated episodes of complete or partial obstruction of the upper respiratory tract during sleep.^1^ Left untreated, OSAS can lead to many medical complications and even death.^2^ The gold standard in OSAS diagnosis is polysomnography (PSG). Studies have demonstrated associations between OSAS and Floppy eyelid syndrome (FES), nonarteritic ischemic optic neuropathy, glaucoma, pseudotumor cerebri and various corneal problems.^3,4,5,6,7,8,9^ OSAS patients have been observed to exhibit FES, papillary conjunctivitis, punctate corneal epitheliopathy, recurrent corneal erosion, keratitis and keratoconus.^3,4,5,9,10^ FES has been the focus of many studies, which attributed the development of dry eye to the inflammatory etiology of OSAS.^4,11^ The aim of this study was to investigate the ocular surface health of patients with OSAS and evaluate their tendency toward dry eyes.

## MATERIALS AND METHODS

Eighty patients who presented to the Gazi University Department of Chest Diseases Sleep Center outpatient clinic for snoring and excessive daytime sleepiness underwent an overnight PSG test and ophthalmological examination. Fifty patients with apnea-hypopnea index (AHI) values over 5 based on their PSG results were included in the study group. Individuals with no complaints like excessive daytime sleepiness, snoring, or obstructive apnea^12^ and no known systemic or ocular diseases were chosen for the control group. The study was approved by the Gazi University Faculty of Medicine Ethics Committee. Aside from apnea, patients in the OSAS group had no systemic or ocular diseases. Twenty-five patients with FES and 5 patients with systemic or ocular disease were excluded from the study. A total of 50 OSAS patients and 50 control subjects were included in the study. OSAS severity was determined based on PSG results as follows: mild, AHI values of 5-15; moderate, AHI values of 15-30; severe, AHI values ≥30.

Before starting continuous positive airway pressure (CPAP) treatment, patients underwent a complete ophthalmological examination including visual acuity assessment, slit-lamp examination, intraocular pressure (IOP) measurement and fundoscopy. In addition to the routine eye exam, patients responded to the ocular surface disease index (OSDI) questionnaire, and their Schirmer I test scores and tear film break-up times (TBUT) were recorded. The study participants’ right eyes were included for both groups.

The OSDI questionnaire consists of three main sections concerning ocular symptoms, visual function and environmental factors.^13^ Each item on the questionnaire is scored from 0 to 4 points. The OSDI score is obtained by summing the points received from the 12 items, multiplying by 25, then dividing by the number of questions answered.^14^

The Schirmer I test was performed by placing a 5x35 mm strip of standard filter paper in the lower eyelid one-third of the distance from the lateral canthus and recording the distance wetted in mm after 5 minutes. TBUT was evaluated by examining the fluorescein-stained tear film with biomicroscope with cobalt blue light and measuring the time between a blink and the first appearance of a dry spot.

All statistical analyses and calculations were done with SPSS version 15.0 (SPSS Inc., Chicago, IL, USA) software package. Descriptive statistics are expressed as mean ± standard deviation. The Shapiro-Wilk test to determine normality of distribution showed the data were not normally distributed. The chi-square test was used in comparisons of qualitative data; the Mann-Whitney U test was used in comparisons of quantitative data. Spearman correlation analysis was used to detect associations between variables. Results of all statistical analyses were evaluated within a 95% confidence interval and p values less than 0.05 were accepted as significant.

## RESULTS

Of the 50 OSAS patients in the study, 39 (78%) were male, 11 (22%) were female and the mean age was 48.01±10.8 (range, 19-68) years. Of the 50 control subjects, 28 (56%) were male, 22 (44%) were female and the mean age was 46.9±12.2 (range, 32-75) years. There were no significant differences between the groups in terms of age or sex (p=0.07, Kruskal-Wallis H test; p=0.06, Pearson chi-square test, respectively). According to AHI scores, OSAS was mild in 15 patients (30%), moderate in 15 patients (30%) and severe in 20 patients (40%) (Table 1).

The mean OSDI scores, TBUTs and Schirmer test values of the OSAS and control groups are presented in Table 2. OSDI score was 18.7±8.5 in the control group versus 40.2±2.8 in mild OSAS, 48.5±2.2 in moderate OSAS and 62.7±2.3 in severe OSAS groups; the difference between groups was significant (p<0.001).

TBUT values were 12.3±4.9 s in the control group and 8.2±4.7 s, 5.8±2.1 s and 4.2±3.7 s in the mild, moderate and severe OSAS groups, respectively; the difference between groups was significant (p<0.001). Schirmer test values were 18±6.1 mm in the control group versus 12.9±6.7 mm, 8.5±5.2 mm and 7.9±4.7 mm in the mild, moderate and severe OSAS groups, respectively; this intergroup difference was also significant (p<0.001).

Body mass index (BMI), AHI severity, and mean oxygen saturation, lowest oxygen saturation and arousal index scores acquired during PSG were significantly correlated with OSDI scores, TBUT and Schirmer test values (p<0.001) (Table 3).

## DISCUSSION

The tear film layer has a complex structure, and normal functioning of the various lacrimal functional unit components is necessary for a healthy tear film layer and ocular surface.^15,16^ Both local and systemic diseases can affect normal tear function and lead to dry eye. The systemic diseases most often accompanied by dry eye are Sjögren’s syndrome and rheumatoid arthritis.^17,18^ Besides autoimmune diseases, dry eye can also be caused by many other systemic diseases including diabetes mellitus, multiple sclerosis and vitamin A deficiency.^19,20^ In the present study we applied the OSDI, Schirmer test and TBUT analysis in order to evaluate the tendency of OSAS patients toward dry eyes.

Dry eye increases with age and is among the diseases that limit daily activities and seriously impact quality of life due to visual impairment and ocular discomfort.^21^ In dry eye, increased tear osmolarity and inflammation cause ocular surface damage.^22^ The lacrimal functional unit consists of the main and accessory lacrimal glands, cornea and conjunctival epithelium, eyelids and meibomian glands; dysfunction in any one of these components can lead to reduced tear production, tear film layer instability and increased tear osmolarity, resulting in ocular surface damage characterized by ocular discomfort and inflammation.^21^ Inflammation plays a key role in the pathogenesis of dry eye, which is an inflammatory disease of the lacrimal glands and ocular surface.^22,23^ The ocular surface inflammation seen in dry eye typically develops as a result of increased tear osmolarity, accumulation of proinflammatory cytokines secreted by the lacrimal glands on the ocular surface and delayed clearing by tears.^21^ In OSAS, levels of proinflammatory cytokines like tumor necrosis factor alpha, interleukin-1 and interleukin-6 are elevated due to chronic intermittent hypoxia.^24^ Cytokines released from dilated conjunctival vessels and damaged epithelium cells create a continual state of inflammation.^25^ As OSAS patients’ AHI increases, so do mechanical tissue stress, hypoxia levels and ocular surface inflammation, which in turn cause meibomian and goblet cell function loss, decreased corneal sensitivity and reduced tear production in response to stimulation to the lacrimal glands.^11^ The loss of meibomian glands and conjunctival goblet cells is reflected clinically as a deterioration in tear film quality.^11^

The OSDI questionnaire is easily applied and is one of the standard methods for symptom assessment and diagnosis in dry eye syndrome.^13,26^ The Turkish translation of the OSDI is reliable^27^ and is currently used in practice and research. In the present study, OSDI scores were significantly higher in the moderate and severe OSAS groups compared to the control group (p<0.001). In the only other study conducted on this topic, Acar et al.^11^ also found significantly higher OSDI scores in the severe OSAS group. In their study, the high OSDI scores were associated with reduced TBUT.^11^ Similarly, we found that all OSAS severity groups exhibited significantly shorter TBUT compared to controls (p<0.016). Mojon et al.^4^ investigated eyelid, conjunctival and corneal findings in 72 OSAS patients and observed reductions in the TBUT of OSAS patients. We also found that Schirmer test results were significantly lower in the moderate and severe OSAS groups compared to the control group (p<0.001). Furthermore, significant positive correlations emerged between OSDI score and both AHI and BMI. As AHI and BMI increased, TBUT and Schirmer values fell significantly.

The assessment of eyelid problems and ocular surface health in OSAS patients has attracted the attention of some researchers. In particular, the frequency of FES in OSAS patients and the pronounced dry eye symptoms that occur in these patients have led to research focusing on OSAS patients with FES.^28,29,30^ Mojon et al.^4^ detected a positive correlation between respiratory distress index and the frequency of FES. Similarly, Acar et al.^11^ found that increasing AHI was positively correlated with frequency of FES. Some studies have reported OSAS and FES coinciding at very low rates (4.5-5%).^30,31^ In order to avoid FES as a confounding factor in our study, we excluded these patients.

This study focused on patients newly diagnosed with OSAS and included only patients who were not being treated with CPAP. Patients undergoing CPAP therapy may experience ocular complications such as dryness and irritation due to air escaping from the mask.^32^ Furthermore, air exposure through the mouth and nose can lead to bacterial conjunctivitis.^33^

A novel aspect of this study is the exclusion of patients with FES, which frequently accompanies OSAS. Even without FES, our results indicate that OSAS patients have a tendency toward dry eyes. Therefore, clinicians should be aware of the possibility of dry eye development in OSAS patients. Studies with larger patient groups are necessary to further our understanding of the pathogenesis of the disease.

## Ethics

Ethics Committee Approval: The study was approved by the Gazi University Faculty of Medicine Ethics Committee, Informed Consent: It was taken.

Peer-review: Externally peer-reviewed.

## Figures and Tables

**Table 1:**
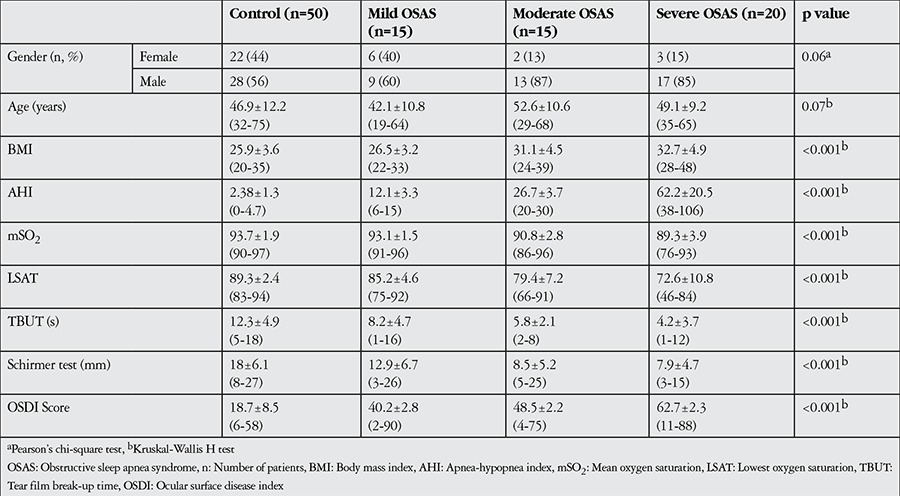
Patients’ demographic, polysomnographic and ophthalmologic characteristics according to apnea-hypopnea index

**Table 2:**
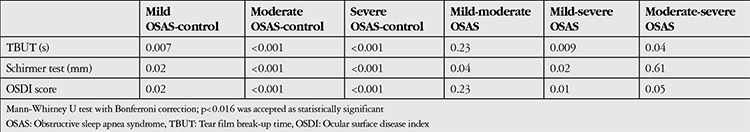
Statistical differences between groups

**Table 3:**
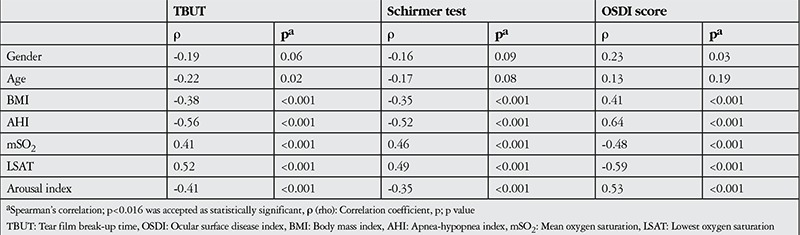
Correlation coefficients and significance values between obstructive sleep apnea syndrome and dry eye parameters
